# The Pedigree of Disease, Being Six Lectures on Temperament, Idiosyncrasy and Diathesis

**Published:** 1886-05

**Authors:** 


**The Pedigree of Disease,** being six lectures on Temperament, Idiosyncrasy, and Diatheses, delivered in the Theatre of the Royal College of Surgeons in the Session of 1881. By JONATHAN HUTCHINSON, F. R. S., Professor of Surgery in the College and in the London Hospital, etc., etc. New York: Wm. Wood & Co., 56 Lafayette Place.

These lectures are from the pen of one of the most learned men of England, excelling in every department of medicine, and possessing an insight into the nature of disease which amounts almost to genius. They are characteristically philosophic, and at the same time so practical that no physician can read them without interest and benefit.

